# Polyphenol Diversity and Chemotype Variation in *Origanum majorana* and Related Species: Implications for Chemotaxonomic Differentiation, Standardisation and Genotype Selection

**DOI:** 10.3390/molecules31091531

**Published:** 2026-05-05

**Authors:** Brigitte Lukas, Johannes Novak, Magdalena Neumüller, Jennifer Romana Valek, Salme Ahmed, Zehra Aytaç, Ahmet Gümüşçü

**Affiliations:** 1 Department of Ecosystem Management, Climate and Biodiversity, Institute of Botany, BOKU University, Gregor-Mendel-Str. 33, 1180 Vienna, Austria; 2 Clinical Department for Farm Animals and Food System Transformation, Nutritional Physiology and Functional Plant Compounds, University of Veterinary Medicine Vienna, Veterinärplatz 1, 1210 Vienna, Austria; johannes.novak@vetmeduni.ac.at (J.N.);; 3 Department of Field Crops, Faculty of Agriculture, Eskişehir Osmangazi University, Ali Numan Kıraç, Campus, 26160 Eskişehir, Turkey; zehrak@ogu.edu.tr (Z.A.); ahmet.gumuscu@ogu.edu.tr (A.G.)

**Keywords:** *Origanum majorana*, polyphenols, phytochemical profiling, chemotype variation, chemotaxonomy, HPLC analysis, genotype selection

## Abstract

This study presents a multivariate assessment of the qualitative and quantitative composition of polyphenolic compounds across six *Origanum* species, including wild and commercial *O. majorana*, the three other species of section *Majorana* (*O. dubium*, *O. syriacum* and *O. onites*), and the more distantly related *O. minutiflorum* and *O. vulgare*. Methanolic extracts from 657 plants representing 59 populations were analysed by HPLC. A total of 122 constituents were consistently detected, 20 major peaks were selected for detailed evaluation, and eight key constituents were quantified using external standards. Wild *O. majorana* was characterised by high proportions of arbutin, apigenin 6,8-di-glucopyranoside, luteolin 7-glucuronide, apigenin 7-glucuronide, rosmarinic acid, salvianolic acid B, blumeatin, and two additional flavonoid-like constituents requiring further structural elucidation. Principal component analysis separated wild and commercial *O. majorana* and distinguished *O. majorana*, the three overlapping clusters of *O. onites*, *O. dubium* and *O. syriacum*, as well as *O. minutiflorum* and *O. vulgare*, reflecting marked interspecific differences in dominant compound classes. *Origanum majorana* and *O. vulgare* were richer in phenolic acids, whereas *O. dubium*, *O. syriacum*, and *O. onites* contained higher levels of flavonoid glycosides. Several genotypes accumulated exceptionally high concentrations of arbutin, apigenin 6,8-di-glucopyranoside, rosmarinic acid, or salvianolic acid B. These results establish a robust chemotaxonomic framework for distinguishing *Origanum* species and pinpoint high polyphenol genotypes as candidates for breeding, quality standardisation, and targeted follow-up studies on antioxidant and other bioactivities.

## 1. Introduction

The genus *Origanum* L. includes 44 species [1,2,3,4,5,6,7], primarily distributed in the Eastern Mediterranean, where they typically inhabit mountainous regions characterised by dry, rocky, and often calcareous soils. Approximately 70 percent of the *Origanum* species are endemic, *O. vulgare* L. is the only species adapted to temperate climate conditions and has a wider distribution across large parts of Europe and Asia [6].

*Origanum* has a complex taxonomy that is complicated by a considerable amount of morphological variation and frequent hybridisation. Among the different taxonomic concepts for *Origanum*, the taxonomic revision by Ietswaart [6], which categorises *Origanum* species into three groups and ten sections, is the most widely accepted. *Origanum majorana* L. (synonym *Majorana hortensis* Moench), an endemic species of Cyprus, is classified alongside three other economically significant *Origanum* taxa, *O. dubium* Boiss., *O. syriacum* L. and *O. onites* L., within the section *Majorana* (group B). According to genetic evidence and the chemical composition of its essential oil, *O. majorana* is a robust taxonomic unit [8,9,10]. Nonetheless, its morphological differentiation from its nearest relatives, *O. dubium* (indigenous to Cyprus and adjacent regions of southern Turkey) and the morphologically diverse *O. syriacum* (native to the Eastern Mediterranean), is challenging and relies on nuanced variations in inflorescences, indumentum and leaves. In particular, the taxonomic status of *O. majorana* and *O. dubium* has long been the subject of discussions [6,7,11,12]. Ietswaart initially classified both under *O. majorana* in his taxonomic revision [6] and in the Flora of Turkey [12] but subsequently reclassified them as distinct species in the Flora of Cyprus [7]. Currently, both the one-species and the two-species concepts are utilised. Cultivated commercial varieties of *O. majorana* (var. *majorana*) have distinct traits compared to the Cypriot wild forms (var. *tenuifolium*) [7]. The commercial types have morphological similarities to *O. syriacum* and *O. dubium*, germinating more rapidly and initiating bud formation considerably earlier than their wild counterparts, with a slightly altered composition of essential oil [10].

The aerial parts of *Origanum* plants are often densely covered with glandular and non-glandular trichomes. The glandular hairs secrete an essential oil with a distinctive scent. Due to these essential oils, *Origanum* species have been harvested or farmed locally for generations for culinary applications and numerous traditional medicinal uses, e.g., [13,14]. Today, marjoram (derived from cultivated *O. majorana*) and oregano (mostly from *O. vulgare* and *O. onites*) are indispensable in the kitchen. Herbal infusions, extracts or creams derived from *Origanum* plant material are utilised for the treatment of upper respiratory tract disorders, gastrointestinal issues or dermatological conditions [14,15]. There exists significant scientific interest in the genus with numerous pertinent publications detailing various pharmacological activities, including antioxidant, antimicrobial, antiparasitic, antifungal, anti-inflammatory, neuroprotective, anticancer, antiproliferative, and cytotoxic activities, e.g., reviewed in [15,16,17]. The diverse beneficial health effects of *Origanum* preparations can be ascribed to an extensive array of secondary metabolites from different chemical groups, including volatile essential oil constituents and polyphenolic compounds; both classes exhibit substantial bioactivity and therefore merit targeted investigation. While essential oils have historically attracted the most attention due to their abundance and aroma-related properties, polyphenolic compounds represent a chemically distinct group with a broad range of strong biological activities and provide complementary insight into the metabolic diversity of the genus. The essential oils of *Origanum* species are often rich in the phenolic monoterpenes carvacrol and thymol (‘cymyl-chemotype’), producing a strong oregano flavour [18,19]. *Origanum majorana* displays an exceptional chemotype within the genus characterised by a near absence of cymyl compounds and a significant accumulation of the bicyclic monoterpenes cis-/trans-sabinene hydrate and cis-sabinene hydrate acetate (‘sabinyl-chemotype’) [8,10], which are responsible for the distinctive ‘marjoramy’ aroma. The presence of a diverse array of non-volatile compounds has been documented, primarily comprising phenolic carbon acids (e.g., caffeic acid, rosmarinic acid, chlorogenic acid, and salvianolic acid), flavones or flavone glycosides (e.g., diverse luteolin- and apigenin-glycosides), hydroquinone derivatives (e.g., arbutin), tannins, triterpenoids (e.g., oleanolic acid and ursolic acid) and sterols (e.g., sitosterol), as described in [15,20,21,22].

Although the beneficial qualities of *Origanum* plant material are becoming more recognised, the natural diversity of the chemicals responsible remains mainly unexplored and chemotaxonomic markers are poorly defined. Pharmacological, methodological or chemical characterisation studies frequently depend on individual trade samples or plant material from one or a limited number of plants or on pooled plant samples. A thorough overview is also obstructed by the challenging intrageneric taxonomic conditions and varying methodological approaches that prohibit direct comparison and synthesis of published findings. Prior research on the natural secondary metabolite diversity of *Origanum* taxa has predominantly concentrated on the analysis of essential oil diversity and the characterisation of essential oil chemotypes in the economically significant *Origanum* species, e.g., [19,23]. Few studies have examined the inter- and intraspecific variability of non-volatile secondary metabolites in *Origanum*. Reference [24] investigated variation in the hydroquinone derivative arbutin across nine *Origanum* species. Studies [25,26] reported pronounced heterogeneity in rosmarinic acid levels within European *O. vulgare*. References [27,28] analysed polyphenolic profiles in a limited number of samples representing four and three subspecies of *O. vulgare*, respectively. Study [29] assessed the chemotaxonomic relevance of exudate flavones and flavanones across nine *Origanum* species. In addition, refs. [30,31] examined polyphenolic compound contents in different harvests of *O. vulgare* and *O. onites* to evaluate the influence of developmental stage and harvest timing on compound composition and concentration.

The primary objective of this investigation was to enhance the limited understanding of polyphenolic compound diversity in *Origanum* using HPLC-based profiling of methanolic extracts from 657 individual plants representing 59 populations and six species. In addition to wild and commercial *O. majorana*, the study also includes *O. dubium*, *O. syriacum*, *O. onites* (all four group B, section *Majorana*), *O. minutiflorum* O. Schwarz & P.H. Davis (group B, section *Chilocalyx*), and *O. vulgare* subsp. *vulgare* (group C, section *Vulgare*). A total of 122 constituents were consistently detected; 20 major peaks were selected for comparative analysis, and several key compounds were quantified using available external standards. Principal component analysis was used to visualise intra- and interspecific polyphenol diversity and to establish a robust chemotaxonomic framework for species and chemotype differentiation. These data highlight high polyphenol genotypes that can inform cultivar development, quality standardisation, and targeted phytochemical or bioactivity studies, and provide chemosystematic insights relevant to pharmacologists and plant breeders.

## 2. Results

A total of 59 accessions (657 individual plants) of seven *Origanum* taxa were analysed. The focus was on wild *O. majorana* (11 wild Cypriot populations, 114 individual plants) as well as commercial *O. majorana* (13 seed origins, 111 individual plants). Furthermore, specimens of *O. dubium* (7 wild Cypriot and Turkish populations, 73 individual plants), *O. minutiflorum* (4 wild Turkish populations, 48 individual plants), *O. onites* (3 wild Turkish populations and 1 seed accession, 33 individual plants), *O. syriacum* (10 Israeli seed accessions and 1 commercial seed origin, 87 individual plants) and *O. vulgare* subsp. *vulgare* (9 Austrian populations, 64 individual plants) were incorporated. Certain specimens from the greenhouse-cultivated populations of commercial *O. majorana* (56 specimens) and *O. syriacum* (35 specimens) were subjected to multiple harvests (fall 2023, early and late summer 2024). All descriptive information concerning species, population sites, seed origins and harvest dates is included in Table S1 of the Supplementary Materials. Of all plants harvested in the flowering stage, plant material of leaves and inflorescences was extracted separately, resulting finally in the analysis of altogether 1185 methanolic extracts (654 leaf extracts and 531 inflorescence extracts).

### 2.1. Extract Composition and Key Components

Following the comparative analysis of typical chromatograms for all included taxa, 122 recurring peaks were identified (Table S2, Supplementary Materials) and further evaluated. Twenty of the 122 peaks had a mean relative peak area exceeding three percent in at least one taxon and were hence classified as main components (Table 1). Among these 20 prominent peaks were one phenolic glycoside (arbutin), five flavonoids (apigenin 6,8-di-glucopyranoside, synonymously known as vicenin II, luteolin 7-diglucuronide, luteolin 7-glucuronide, apigenin 7-glucuronide, and blumeatin) and two phenolic acids (rosmarinic acid and salvianolic acid B). Four more flavonoid compounds (peak 44a, 45a, 88 and 95) have yet to be determined; one other compound was tentatively recognised as the lignan globoidnan A. In addition to the characteristic polyphenols, the methanolic extracts also contained the structurally related yet non-polyphenolic phenolic monoterpenes thymol and carvacrol, which appear in the HPLC chromatograms due to their comparable polarity. Among the minor compounds (mean relative peak area of less than three percent across all seven taxa) were hydroquinone and an unidentified hydroquinone derivative, along with other flavonoid glycosides (apigenin 8-glucoside syn. vitexin, hesperetin 7-rutinoside syn. hesperidin, and apigenin 7-glucoside), flavonoid aglycons (eriodictyol, naringenin, and apigenin), and phenolic acids (caffeic acid and lithospermic acid) (Table S2, Supplementary Materials).

**Figure 1:**
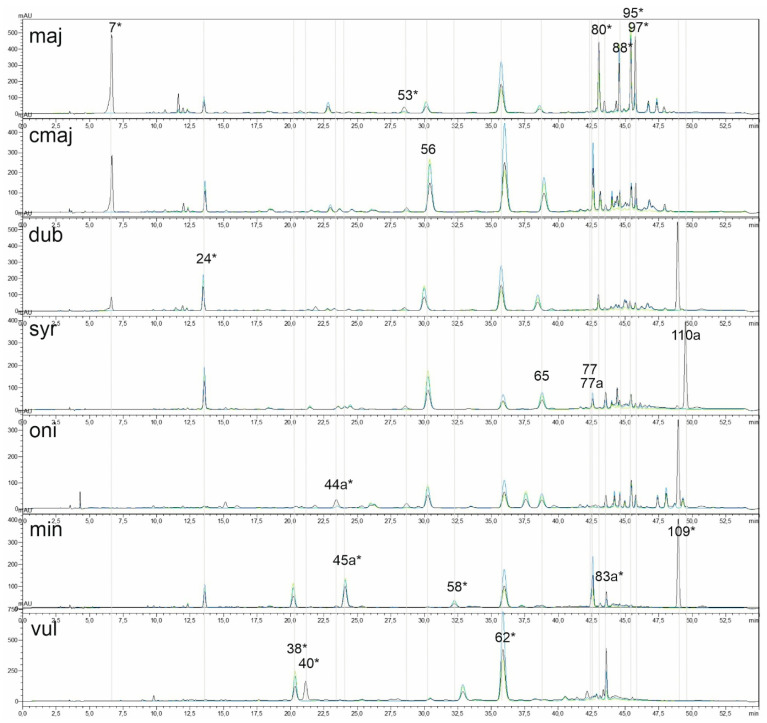
Representative HPLC chromatograms (UV at 280, 330, and 345 nm) for the seven taxa: maj = *O. majorana*, cmaj = commercial *O. majorana*, dub = *O. dubium*, syr = *O. syriacum*, oni = *O. onites*, min = *O. minutiflorum*, vul = *O. vulgare*. Chromatograms are ordered by relatedness to *O. majorana* (top to bottom). Peak numbers correspond to Table 1 and are shown in the taxon with the highest mean area for each peak. Line colours: 280 nm (black), 330 nm (blue), and 354 nm (green). PCA variables are marked \*.

Qualitatively, the chromatograms of all six species exhibited stable and, in the case of *O. majorana*, *O. minutiflorum* and *O. vulgare*, also quite differentiated peak patterns (Figure 1). The extracts of wild Cypriot *O. majorana* were characterised by a significant arbutin peak (peak 7) at the beginning of the chromatogram, rosmarinic acid (peak 62) and a unique group of four closely spaced components in the latter half of the chromatogram (RT 43 min to RT 47 min) comprising salvianolic acid B (peak 80), two unidentified flavonoid compounds (peaks 88 and 95) and blumeatin (peak 97). Commercial *O. majorana* exhibited differences from the wildtype, characterised by a diminished arbutin peak, an amplified rosmarinic acid peak, and a markedly less prominent peak group between RT 43 min and RT 47 min. The chromatograms of *O. minutiflorum* were distinguished by the distinctive presence of peak 45a, peak 58, globoidnan A (peak 83a) and carvacrol (peak 109), while the chromatograms of *O. vulgare* subsp. *vulgare* were characterised by the co-occurrence of luteolin 7-diglucuronide (peak 38), peak 40 and a significant peak of rosmarinic acid (peak 62). The chromatograms of *O. dubium*, *O. syriacum* and *O. onites*, which lacked distinct individual peak patterns, were characterised by a relatively large apigenin 6,8-di-glucopyranoside peak (peak 24, *O. dubium* and *O. syriacum*) or a notably prominent peak 44a (exclusive to *O. onites*), along with a significant carvacrol peak (peak 109, for *O. syriacum* a carvacrol and/or a thymol (peak 110a)) at the end of the chromatogram. The two components, luteolin 7-glucuronide (peak 56) and apigenin 7-glucuronide (peak 65), were a common characteristic of the four species of the *Majorana* section.

**Figure 2:**
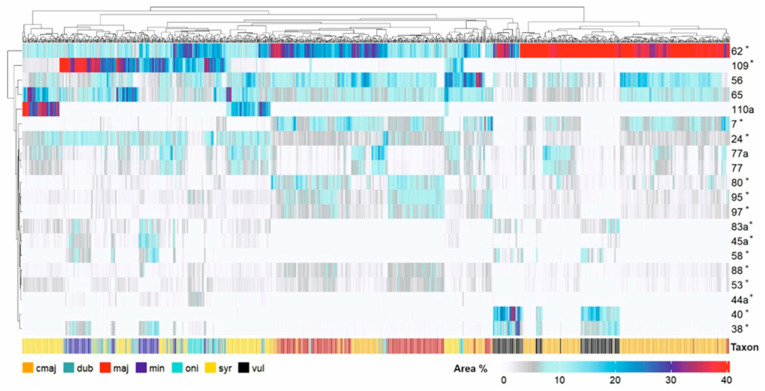
Clustered heatmap (Euclidean distance) of relative area percentages for the 20 main components across all extracts. Rows = peak numbers (see Table 1). Colour legend indicates relative area (%). Taxa: maj = *O. majorana*, cmaj = commercial *O. majorana*, dub = *O. dubium*, syr = *O. syriacum*, oni = *O. onites*, min = *O. minutiflorum*, vul = *O. vulgare*. PCA variables are marked \*.

A clustered heatmap utilising all extracts and the 20 main components (Figure 2) categorised the samples into two primary clusters. The diminutive cluster at the right side of the heatmap was distinguished by a substantial concentration of rosmarinic acid (peak 62) and encompassed the majority of commercial *O. majorana* samples, along with several wild *O. majorana* samples, which were aggregated with two integrated sub-clusters comprising *O. vulgare* subsp. *vulgare* samples (readily identifiable by the dark-coloured columns in the rows corresponding to luteolin 7-diglucuronide (peak 38) and peak 40). In the larger cluster on the left, samples of *O. majorana*, *O. dubium*, *O. syriacum*, *O. onites*, and *O. minutiflorum* did not establish distinct, taxon-specific clusters, but rather formed smaller, mixed subclusters. On the right side of the larger cluster, the majority of the samples were wild *O. majorana* from Cyprus, clustered with commercial *O. majorana* (identifiable by the darker lines in the rows corresponding to peaks 77 and 77a), a secondary group of *O. vulgare* samples, along with some *O. onites* samples (darker stained in the row corresponding to peak 44a) and *O. syriacum* samples (darker stained in the row corresponding to carvacrol (peak 109)). The sub-cluster comprising the wild Cypriot *O. majorana* samples and a subset of the commercial *O. majorana* was distinctly delineated by the pronounced presence of salvianolic acid B (peak 80), peak 95 and blumeatin (peak 97). Moreover, nearly all wild and commercial *O. majorana* specimens exhibited the lack of eight specific components: luteolin 7-diglucuronide (peak 38), peak 40, peak 44a, peak 45a, peak 58, globoidnan A (peak 83a), carvacrol (peak 109), and thymol (peak 110a).

### 2.2. Sample Classification

**Figure 3:**
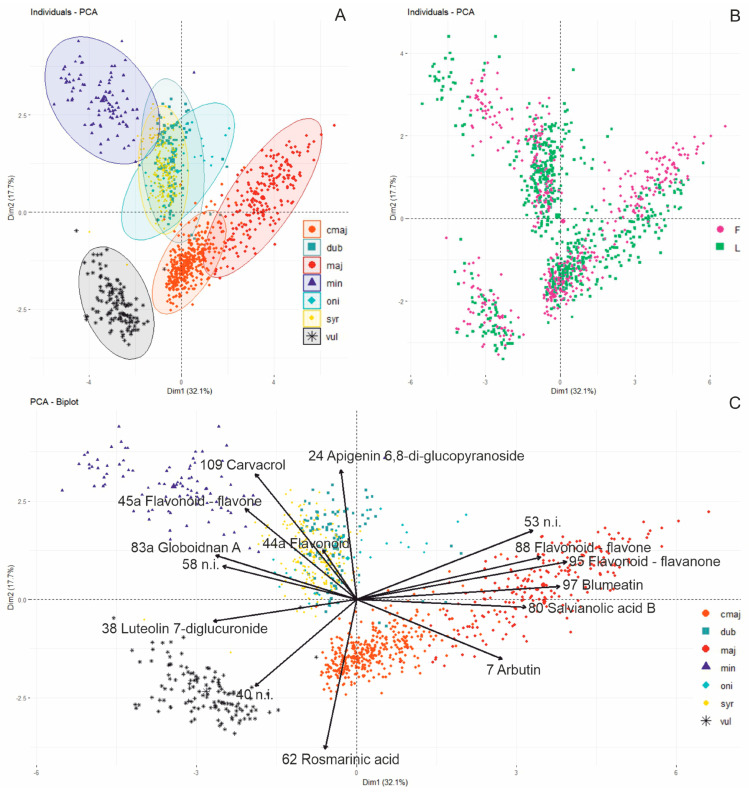
Principal component analysis (PCA) based on the 15 main components selected by cos^2^. (**A**) Scores plot (Dim 1 vs. Dim2) for all samples and taxa. (**B**) Scores plot separated by tissue type: inflorescences (F) and leaves (L). (**C**) Variable factor loadings showing each component’s contribution to the PCA dimensions. N.i. denotes compounds that have not yet been identified. Taxa: maj = *O. majorana*, cmaj = commercial *O. majorana*, dub = *O. dubium*, syr = *O. syriacum*, oni = *O. onites*, min = *O. minutiflorum*, vul = *O. vulgare* subsp. *vulgare*.

The comprehensive findings of the HPLC chromatogram analysis (1185 extracts, relative area percentages of 122 peaks) were illustrated by principal component analysis (PCA). The first two dimensions revealed a distinctly separated cluster of *O. vulgare* samples adjacent to the largely overlapping clusters of *O. minutiflorum*, *O. majorana*, *O. onites*, *O. dubium*, and *O. syriacum*, demonstrating a minimal overall explanatory power of 17% (figure not displayed). The squared cosine (cos^2^) values were utilised to find the 30 variables with the greatest discriminatory power based on the first four dimensions (Table S2, Supplementary Materials). Fifteen of the 20 major components listed in Table 1, specifically arbutin, apigenin 6,8-di-glucopyranoside, luteolin 7-diglucuronide, peak 40, peak 44a (flavonoid), peak 45a (flavonoid), peak 53, peak 58, rosmarinic acid, salvianolic acid B, globoidnan A, peak 88 (flavonoid), peak 95 (flavonoid), blumeatin and carvacrol, were among these variables and were subsequently employed for PCA utilising a reduced set of variables. The initial two dimensions of this simplified PCA accounted for approximately 50% of the observed variance (Figure 3A). Principal component 1 (32%) distinctly separated *O. syriacum*, *O. minutiflorum* and *O. vulgare* from the wild *O. majorana*, while principal component 2 (18%) divided *O. minutiflorum*, *O. dubium*, *O. syriacum*, and *O. onites* from *O. vulgare* and commercial *O. majorana*. The samples of *O. minutiflorum*, *O. vulgare* and *O. majorana* each formed different clusters. The samples of commercial *O. majorana* and the wild Cypriot *O. majorana* could be distinctly differentiated. As already seen in the heat map (Figure 2), commercial *O. majorana* exhibited similarities to *O. vulgare* subsp. *vulgare*. The samples of *O. onites*, *O. syriacum* and *O. dubium* were indistinguishable from one another; however, the integration of dimensions 1, 2 and 3 with dimension 4 facilitated the distinguishing of at least some *O. onites* samples (figure not displayed). Despite integrating data from floral and foliar samples (*O. syriacum*, *O. onites*, *O. dubium*, *O. minutiflorum*, *O. vulgare*, and *O. majorana*) and samples harvested across various seasons (*O. syriacum* and commercial *O. majorana*), the corresponding taxon clusters exhibited considerable compactness, with minimal scatter. Solely in wild *O. majorana*, leaf and inflorescence samples tended to cluster separately (Figure 3B). The characteristics that distinctly differentiate *O. majorana* include arbutin, peak 53, rosmarinic acid, salvianolic acid B, peak 88 (a flavonoid), and blumeatin (Figure 3C). Rosmarinic acid predominantly influenced the distinction between commercial and wild *O. majorana*. The primary variables contributing to the distinction of *O. minutiflorum*, *O. onites*, *O. dubium* and *O. syriacum* were the compounds apigenin 6,8-di-glucopyranoside, peak 44a (flavonoid), peak 45a (flavonoid), peak 58, globoidnan A and carvacrol. The primary determinants for the differentiation of *O. vulgare* were luteolin 7-diglucuronide and peak 40.

### 2.3. Total Contents of Selected Key Components

**Figure 4:**
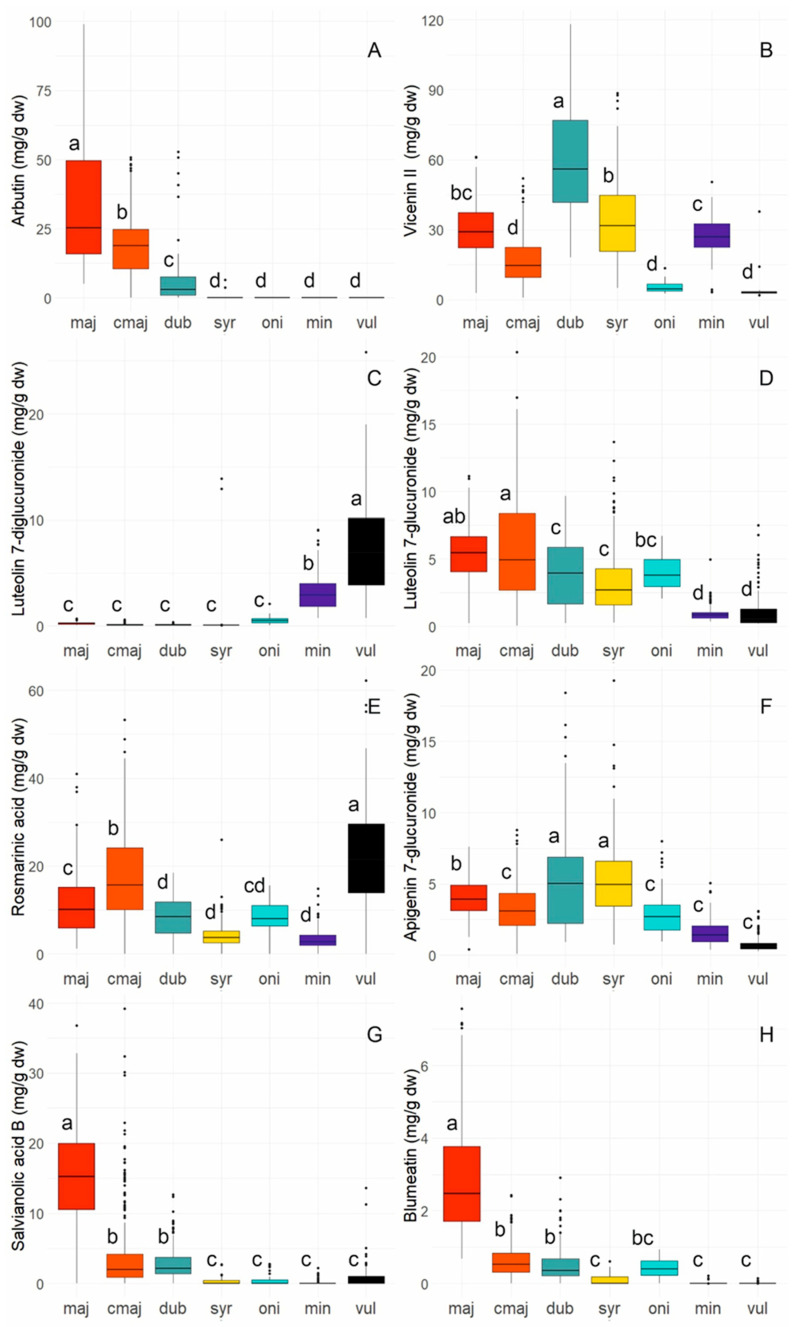
Quantitative comparison (mg/g dry wt) of selected compounds across taxa. Panels: (**A**) arbutin, (**B**) apigenin 6,8-di-glucopyranoside (syn. vicenin II), (**C**) luteolin 7-diglucuronide, (**D**) luteolin 7-glucuronide, (**E**) rosmarinic acid, (**F**) apigenin 7-glucuronide, (**G**) salvianolic acid B, (**H**) blumeatin. Taxa: maj = *O. majorana*, cmaj = commercial *O. majorana*, dub = *O. dubium*, syr = *O. syriacum*, oni = *O. onites*, min = *O. minutiflorum*, vul = *O. vulgare* subsp. *vulgare*. Different letters above bars indicate statistically distinct groups. (Tukey HSD, α = 0.005).

To illustrate the occurrence and variability of the eight clearly identified major polyphenolic components: arbutin, apigenin 6,8-di-glucopyranoside, luteolin 7-diglucuronide, luteolin 7-glucuronide, rosmarinic acid, apigenin 7-glucuronide, salvianolic acid B, and blumeatin, their peak areas were quantified and graphically represented (Figure 4; a comparative analysis of the contents of leaf extracts and flower extracts is provided in the Supplementary Materials, Figure S1).

#### 2.3.1. Phenols and Phenolic Glycosides

Hydroquinone, the aglycon of arbutin, was consistently detected in the samples, albeit at minimal relative percentages (Table S2, Supplementary Materials). Substantial quantities of arbutin were exclusively identified in wild *O. majorana* (median content (Q50) 25 mg/g dry weight, maximum content (max) 99 mg/g dry weight), commercial *O. majorana* (Q50 19 mg/g, max 51 mg/g), and *O. dubium* (Q50 3 mg/g, max 53 mg/g) (Figure 4A). Wild *O. majorana*, commercial *O. majorana,* and *O. dubium* exhibited considerable differences from one another and from all other *Origanum* species in which arbutin was not discovered at all. In *O. majorana* and *O. dubium*, arbutin formed a close double peak with a second potential hydroquinone derivative (peak 6). In wild *O. majorana*, where elevated levels of both compound 6 and arbutin were observed, the ratio of the presumed second hydroquinone derivative to arbutin appeared rather stable at approximately 1:3.

#### 2.3.2. Flavonoids and Flavonoid Glycosides

The four primary flavonoid glycosides identified were derivatives of apigenin and luteolin. Apigenin was classified as a minor constituent, but luteolin was not distinctly identifiable in our extracts (Table S2, Supplementary Materials). Apigenin 6,8-di-glucopyranoside, also known as vicenin II, was the most significant flavonoid glycoside quantitatively (Figure 4B). In *O. dubium*, the median content (Q50) of apigenin 6,8-di-glucopyranoside was 56 mg/g dry weight (maximum content 118 mg/g dry weight). *Origanum dubium* exhibited significant differences from *O. syriacum* (Q50 32 mg/g, max 89 mg/g), *O. minutiflorum* (Q50 27 mg/g, max 50 mg/g), commercial *O. majorana* (Q50 15 mg/g, max 52 mg/g), *O. onites* (Q50 5 mg/g, max 14 mg/g), and *O. vulgare* (Q50 3 mg/g, max 38 mg/g). Wild *O. majorana* (Q50 29 mg/g, max 61 mg/g) could not be distinctly separated from *O. syriacum* and *O. minutiflorum*. Luteolin 7-diglucuronide (Figure 4C) was a character compound of *O. vulgare* subsp. *vulgare* (Q50 7 mg/g, max 26 mg/g) and also present, albeit with lesser significance, in *O. minutiflorum* (Q50 3 mg/g, max 9 mg/g). *Origanum vulgare* and *O. minutiflorum* differed significantly from one another and from all other taxa, which often contained only negligible quantities of this compound. Intermittently, elevated concentrations of luteolin 7-diglucuronide were detected in *O. syriacum* (Q50 3 mg/g, max 14 mg/g) and *O. onites* (Q50 4 mg/g, max 7 mg/g). Species accumulated elevated levels of luteolin 7-diglucuronide or luteolin 7-glucuronide (Figure 4C,D). The greatest concentrations of luteolin 7-glucuronide (Figure 4D) were found in commercial *O. majorana* (Q50 5 mg/g, max 20 mg/g). Wild *O. majorana* (Q50 6 mg/g, max 11 mg/g) could not be distinctly differentiated from commercial *O. majorana* and *O. onites* (Q50 4 mg/g, max 7 mg/g). The luteolin 7-glucuronide content in *O. onites* was similar to that in *O. dubium* (Q50 4 mg/g, max 10 mg/g) and *O. syriacum* (Q50 3 mg/g, max 14 mg/g), which significantly differed from *O. vulgare* (Q50 < 1 mg/g, max 8 mg/g) and *O. minutiflorum* (Q50 < 1 mg/g, max 5 mg/g). The overall concentrations of luteolin 7-glucuronide and apigenin 7-glucuronide were approximately equivalent. The largest median concentrations of apigenin 7-glucuronide were observed in *O. syriacum* (Q50 5 mg/g, max 19 mg/g) and *O. dubium* (Q50 5 mg/g, max 18 mg/g). The two species exhibited significant differences from wild *O. majorana* (Q50 4 mg/g, max 8 mg/g), as well as from commercial *O. majorana* (Q50 3 mg/g, max 9 mg/g), *O. onites* (Q50 3 mg/g, max 8 mg/g), *O. minutiflorum* (Q50 1 mg/g, max 5 mg/g), and *O. vulgare* (Q50 < 1 mg/g, max 3 mg/g). The accumulation of the flavanone blumeatin seems to be a characteristic of wild *O. majorana* (Q50 3 mg/g, max 8 mg/g). Commercial *O. majorana* (Q50 < 1 mg/g, max 2 mg/g) and *O. dubium* (Q50 < 1 mg/g, max 3 mg/g) sporadically demonstrated elevated levels of blumeatin. Neither taxon could be meaningfully distinguished from *O. onites* (Q50 < 1 mg/g, max < 1 mg/g), but they could be discriminated from *O. syriacum* (Q50 0 mg/g, max < 1 mg/g), *O. minutiflorum* (Q50 0 mg/g, max < 1 mg/g), and *O. vulgare* (Q50 0 mg/g, max < 1 mg/g). In the latter four species, the diminutive blumeatin peak was typically partially obscured by a subsequent minor peak, complicating the verification of its presence.

#### 2.3.3. Phenolic Acids

Caffeic acid, the fundamental phenylpropanoid unit of the more intricate rosmarinic acid and the salvianolic acids, was consistently detected among the minor compounds (Table S2, Supplementary Materials). Rosmarinic acid (Figure 4E) typically constituted one of the most significant peaks in the chromatograms. The highest concentrations were observed in *O. vulgare* (median content (Q50) 22 mg/g dry weight, maximum (max) concentration 62 mg/g dry weight), which were significantly distinct from commercial *O. majorana* (Q50 16 mg/g, max 53 mg/g), which in turn displayed a notably higher median content of rosmarinic acid than wild *O. majorana* (Q50 10 mg/g, max 41 mg/g). *Origanum onites* (Q50 8 mg/g, max 16 mg/g) could not be distinguished from wild *O. majorana*; however, *O. dubium* (Q50 8 mg/g, max 18 mg/g), *O. syriacum* (Q50 4 mg/g, max 26 mg/g), and *O. minutiflorum* (Q50 3 mg/g, max 15 mg/g) exhibited significant differences. Salvianolic acid B (Figure 4G), like arbutin and blumeatin, was a characteristic compound of wild *O. majorana* (Q50 15 mg/g, max 37 mg/g). The salvianolic acid B concentration in wild *O. majorana* considerably differed from that in commercial *O. majorana* and *O. dubium* (both with a Q50 of 2 mg/g dry), which occasionally displayed greater levels of salvianolic acid B (max 13 mg/g in *O. dubium,* max 39 mg/g in commercial *O. majorana*). *Origanum syriacum*, *O. onites*, *O. minutiflorum* and *O. vulgare* typically contained very negligible quantities of salvianolic acid B (Q50 below 1 mg/g).

#### 2.3.4. Comparison of Inflorescences and Leaves

When comparing the plant material from inflorescences and leaves, the arbutin content of the leaf material was significantly higher in wild *O. majorana* and tended to be higher in commercial *O. majorana* and *O. dubium* as well (Figure S1A, Supplementary Materials). No general tendencies could be observed between the different species regarding phenolic acids, flavonoids or flavonoid glycosides (Figure S1, Supplementary Materials).

## 3. Discussion

Methanolic extracts from the leaves and inflorescences of 657 individual plant samples across six *Origanum* species were analysed via HPLC-DAD to establish a preliminary inventory of natural polyphenol variability within wild and commercial *O. majorana,* and to assess its specific qualitative and quantitative extract composition in direct comparison with closely related species (*O. dubium*, *O. syriacum*, and *O. onites*) and more distantly related species (*O. minutiflorum* and *O. vulgare* subsp. *vulgare*).

A total of 122 recurring peaks were defined, and their presence and peak areas were documented. Twenty of the 122 components reached a mean relative peak area percentage above three in at least one species and were classified as main components. To date, ten of these main components have been identified and validated through comparison with reference chromatographic standards. Among these ten main constituents were a phenolic glycoside (arbutin), flavonoids and flavonoid glycosides (apigenin 6,8-di-glucopyranoside, luteolin 7-diglucuronide, luteolin 7-glucuronide, apigenin 7-glucuronide, and blumeatin), two phenolic acids (rosmarinic acid and salvianolic acid B), and the phenolic monoterpenes carvacrol and thymol. Another phenolic compound, the lignan globoidnan A, was recognised by its UV spectrum [32], while four other compounds, categorised as undeclared flavonoids, were provisionally identified based on spectrum shape and UV absorption maxima (Table 1).

Higher concentrations of arbutin were found in *O. majorana* and moderate levels in *O. dubium*, while several other *Origanum* species, including *O. syriacum*, *O. onites*, and *O. vulgare* subsp. *vulgare*, lacked detectable arbutin, as previously documented [24]. The arbutin contents reported here tended to be lower than those in [24] (same extraction medium but different plant material:solvent ratio) and were approximately comparable to values reported for commercial *O. majorana* water extracts [33,34]. Arbutin is a glucoside of hydroquinone and can be metabolically or chemically converted to free hydroquinone under certain conditions; the free aglycone is considered toxicologically relevant. However, human exposure from typical culinary use of marjoram is low: using common culinary amounts (0.2–0.3 g dried herb per dish [24]) and the mean arbutin content reported here for commercial marjoram (2% *w*/*w*), the estimated intake per dish would be on the order of 4–6 mg arbutin. Dietary exposures to arbutin/hydroquinone from common foods and herbal preparations have generally been considered of low concern under recommended use conditions, although high or chronic exposures and concentrated extracts may warrant further evaluation and standardisation. The persistent occurrence of a second hydroquinone derivative in *O. majorana* and *O. dubium* (peak 6, a minor compound with an average area percentage of 2.5% in wild *O. majorana*) was validated by an alternative extraction method involving acidic hydrolysis. Robustaside B has previously been isolated together with arbutin from Hungarian *O. majorana* [35], and seguinoside B, as well as osmantolide, have been reported from Cypriot *O. dubium* [36]. However, because analytical reference standards for these candidate compounds are not available, the identity of the unknown hydroquinone derivative could not be confirmed. Structural confirmation will require isolation and complementary analyses (targeted LC-MS/MS including MS/MS fragmentation and high-resolution MS, and/or NMR) or comparison with authentic standards; we propose this as a priority for future work.

Quantitatively, apigenin 6,8-di-glucopyranoside (syn. vicenin II) was the predominant flavonoid in our extracts. This compound has been identified in various aqueous, ethanolic, and methanolic extracts of *O. majorana* [22,37,38,39], *O. syriacum* [15,40], *O. minutiflorum* [41,42], and *O. vulgare* [30,43] (both subsp. *hirtum*), but, to our knowledge, it has not been found in *O. dubium*, the species with the highest total concentration of apigenin 6,8-di-glucopyranoside (up to 118 mg/g). Luteolin 7-diglucuronide has been described as a major constituent of aqueous extracts from *O. vulgare* subsp. *vulgare* [44] but not for *O. minutiflorum*, the sole other *Origanum* species examined here, which exhibited elevated levels of this compound. Luteolin 7-glucuronide and apigenin 7-glucuronide have been previously identified in aqueous or methanolic extracts of *O. majorana*, e.g., [37,38,39]; *O. minutiflorum* (solely apigenin 7-glucuronide) [41]; and *O. vulgare* [27,30]. The quantified amounts of apigenin 6,8-di-glucopyranoside and luteolin 7-glucuronide in [45] and [30] are almost equivalent to the quantified values obtained in this study for *O. vulgare* and for *O. majorana*, respectively. Blumeatin has been discussed as a distinctive constituent of *O. majorana* [46], albeit this assertion can only be partially substantiated. Wild and commercial *O. majorana* differed significantly in their blumeatin content, with elevated levels seen solely in the wild variant. Apigenin 7-glucoside [30], apigenin 8-glucoside (syn. vitexin) [15,22], hesperetin 7-rutinoside (syn. hesperidin) [22], and the flavonoid aglycons apigenin, e.g., [37,38,41,42,43,47], eriodictyol [22,29,41], and naringenin [29,39,48] were additionally identified as flavonoids among the recorded minor compounds (Table S2, Supplementary Materials). Several previously identified *Origanum* flavonoid derivatives or flavonoid aglycons, including quercetin 3-rhamnoside [39,49], naringenin 7-neohesperidoside (syn. naringin) [31,50], hesperetin [45,48], luteolin, e.g., [22,41,43], and catechin/epicatechin [28,43,48,50], could not be distinctly identified in our extracts.

Rosmarinic acid is often regarded as the predominant phenolic acid in aqueous and methanolic extracts of *O. majorana*, e.g., [37,38,48], *O. syriacum* [25], *O. onites* [25,31,49], *O. minutiflorum* [41,42], and *O. vulgare*, e.g., [27,30,43]. The quantified amounts reported here are comparable to those found in Israeli *O. syriacum*, Turkish O. *onites,* and *O. vulgare* collected across Europe [25] (same extraction medium but different plant material:solvent ratio), as well as to Austrian and Italian *O. vulgare* (methanolic extracts) [26,50] and to *O. vulgare* subsp. *hirtum* cultivated in pots in Denmark (methanolic extracts, second season) [30]. They are, however, considerably higher than values reported for methanolic extracts of certain Iranian *O. vulgare* specimens [28]. Rosmarinic acid levels vary markedly across studies because of multiple, often interacting factors. Besides genetic differences [27], variation may also arise from geographical origin and local edaphic and climatic conditions, agronomic practices and phenological stage at harvest, the plant part analysed, and differences in analytical and pre-analytical procedures. Consequently, direct quantitative comparisons between different studies should be interpreted with caution unless sampling and analytical conditions are closely matched. Salvianolic acid B has previously been reported in *O. majorana* [37,45] and *O. vulgare* subsp. *vulgare* and subsp. *hirtum* [44,51,52]. In this study, appreciable amounts of salvianolic acid B were detected only in *O. majorana*. The quantities measured here are comparable to those reported in [45]. Globoidnan A, categorised as a lignan yet structurally a tetramer of caffeic acid, has previously been reported for *O. minutiflorum* [41] but, to our knowledge, not for *O. syriacum* and *O. vulgare*. Caffeic acid, e.g., [31,39,43], a building block of rosmarinic acid, the salvianolic acids, and globoidnan A, as well as lithospermic acid A [37,44], were additional phenolic carbon acids identified among the minor compounds (Table S2, Supplementary Materials). The phenolic acids previously reported from *Origanum* species, chlorogenic acid [22,39,48], ferulic acid [48,53], gallic acid [28,48,50], salvianolic acid A [52], and salvianolic acid C [27,48], could not be definitively verified in our extracts.

Principal component analysis of the relative area percentages of 15 of the 20 major components revealed that *O. majorana*, *O. minutiflorum* and *O. vulgare* were largely distinguishable from each other and from *O. dubium*, *O. syriacum* and *O. onites,* which exhibited significant overlap in an intermediate cluster. The clear differentiation of *O. majorana* was predicated on arbutin, peak 53, rosmarinic acid, salvianolic acid B, peak 88 (flavonoid), peak 95 (flavonoid) and blumeatin. The elevated levels of the strongly positively correlated compounds 53, salvianolic acid B, peak 88, peak 95 and blumeatin distinctly differentiated wild *O. majorana* from the analysed commercial *O. majorana* accessions and may serve as chemotaxonomic markers for the delineation of the *O. majorana* wildtype. In turn, commercial *O. majorana* had a markedly elevated content of rosmarinic acid. The other three species of section *Majorana*, *O. dubium*, *O. syriacum* and *O. onites,* were distinguished by apigenin 6,8-di-glucopyranoside, compound 44a, and carvacrol. Carvacrol, along with compounds 45a, 85 and 83a, was a significant variable for the distinct clustering of the *O. minutiflorum* samples. *Origanum vulgare* subsp. *vulgare* could be distinctly differentiated by peak 40 and luteolin 7-diglucuronide. PCA thus enabled a clear differentiation between the samples of *Origanum* group C (section *Origanum*) and those of *Origanum* group B (the sections *Chilocalyx* and *Majorana*) as well as a substantial separation among the species of sections *Origanum* (*O. vulgare*), *Chilocalyx* (*O. minutiflorum*) and *Majorana* (*O. majorana*, *O. dubium, O. syriacum* and *O. onites*).

The notable aspect is the considerable differentiation between *O. majorana* and the other three species of section *Majorana*, namely *O. dubium*, *O. syriacum* and *O. onites*, which exhibit significant overlap in their polyphenol characteristics. A comparable pattern is evident when comparing their composition of essential oil compounds. *Origanum majorana* possesses a unique essential oil chemotype within the section, characterised by a high percentage of sabinyl compounds that impart the distinctive ‘marjoramy’ flavour [8,54], whereas the essential oils of *O. onites*, *O. dubium* and *O. syriacum* are typically abundant in cymyl compounds characteristic of oregano [18,19,55]. Taxonomically, section Majorana presents challenges due to its inclusion of four morphologically similar species: the morphologically somewhat better-defined *O. onites* alongside *O. majorana*, *O. dubium* and *O. syriacum,* which can only be differentiated by nuanced variations in inflorescence and leaf morphology and indumentum [6,7,12]. Genetically, all four *Majorana* species display distinct characteristics that affirm the more remote status of *O. onites* and suggest a close genetic relationship between *O. majorana* and *O. syriacum*, a cryptic hybridogeneous origin of *O. dubium*, a somewhat intermediate position of commercial *O. majorana* relative to wild *O. majorana* and *O. syriacum*, and recent hybridisation of sympatric *O. onites* and *O. dubium* in Turkey [9]. The spectra of polyphenol and essential oil constituents distinctly delineate *O. majorana* within section *Majorana*, a distinction not as strongly reflected in the morphological traits and the published DNA sequence and SSR data to date. Another striking result is the distinct differentiation between wild Cypriot and commercial *O. majorana* accessions, which occupy an intermediate position between wild *O. majorana* and *O. vulgare*, with some samples tending towards the overlapping species clusters of *O. onites*, *O. dubium* and *O. syriacum*. Phenological and morphological distinctions between wild and commercial *O. majorana*, as earlier flowering of the cultivars and subtle differences in leaf shape and leaf colour and also in essential oil chemistry have already been documented [10]. The ramifications of centuries of selective breeding and/or hybridisation in the cultivation history of the commercial plants may account for this outcome. In European regions where marjoram is commercially grown for herb and seed production, genetic exchange with the native species *O. vulgare* may occasionally occur. Nevertheless, despite the significantly increased rosmarinic acid contents, the extracts from the commercial marjoram plants showed no direct evidence of genetic exchange with *O. vulgare*. Compounds 77 and 77a, absent in wild *O. majorana* but present in *O. dubium* and *O. syriacum*, suggest a connection between commercial *O. majorana* and these two species. Besides genetic variables, ecological factors associated with latitude may also play a role. All analysed accessions of commercial *O. majorana* were cultivated in the Central European climate from randomly selected, locally sourced commercial seeds (cmaj03–cmaj12; Table S1, Supplementary Materials) or seeds from hybrid varieties of a previous breeding project (cmaj15–cmaj19, [56,57]). It is yet to be determined whether commercial *O. majorana* from a Mediterranean climate or different seed lines demonstrates similar qualitative and quantitative characteristics in its polyphenolic compound composition as the thirteen marjoram seed accessions investigated herein. *Origanum majorana* s. str. and *O. dubium* have historically been classified together under *O. majorana* s.l. [6,12]. Their close morphological relationship is not immediately evident in the PCA; however, similar to commercial *O. majorana*, *O. dubium* also demonstrated relatively low levels of arbutin, salvianolic acid B and blumeatin—compounds recognised as characteristic constituents of wild *O. majorana*. The concurrent presence of these within the genus’s uncommon secondary metabolites may indicate a shared ancestor of *O. dubium* and *O. majorana* or suggest a role of *O. majorana* in the obscure speciation history of *O. dubium*. Hybridisation can result in the reorganisation of metabolic pathways and the synthesis of novel compounds [58]. Recent hybridisation may be expressed in the differentiation of the *O. onites* population from the Gulf of Antalya [59], where local hybridisation between *O. onites* and *O. dubium* is occurring [9].

This investigation utilised a broad sample set encompassing plant material from various species and populations, from both wild habitats and cultivated pots, from Mediterranean and temperate climates, from diverse phenological stages (predominantly flowering, some in seed development), from different harvest times (late autumn, early summer, and late summer), and from distinct plant organs (inflorescences and leaves). Notwithstanding numerous potentially local or temporal factors affecting sample secondary compound chemistry, the PCA (utilising the percentages of 15 key components) revealed notably compact and, for three species, distinctly defined clusters that align with taxonomic classifications based on morphological traits [6,7] and patterns identified through comparative essential oil analysis [8,10,18]. The PCA clusters of *O. majorana*, *O. minutiflorum* and *O. vulgare* subsp. *vulgare*, along with the cluster comprising *O. dubium*, *O. syriacum*, and *O. onites*, signify four distinct polyphenol chemotypes characterised not only by varying percentages of co-distributed compounds but also by consistent section- or species-specific compound profiles. The noticeably homogenous plant material from the various seed accessions of commercial *O. majorana* and Israeli *O. syriacum* (both taxa represented by leaf and inflorescence samples from three different harvests) cultivated concurrently under Viennese climatic conditions would substantiate our hypotheses that the four observed chemotypes are predominantly influenced by genetic characteristics of species or higher taxa. Secondary metabolites are characterised as stable and reliable biomarkers capable of differentiating or identifying species and even intraspecific taxa [60,61]. Ref. [62] delineates a pronounced species specificity, distinct geographic structure and strong genetic determination of flavonoid abundances in *Populus* and hypothesises relaxed selective pressures in the more downstream portions of the phenylpropanoid pathway. Moreover, their findings indicate a potentially significant role of quickly evolving glycosyltransferase enzymes in the diversity and interspecific differentiation they observed. These considerations align effectively with the outcomes obtained herein. Rosmarinic acid, apigenin glycosides and luteolin glycosides were widely distributed, whereas more complex caffeic acid oligomers or derivatives (salvianolic acid B and globoidnan A), specific flavonoid glycosides (apigenin 6,8-di-glucopyranoside, luteolin 7-diglucuronide, and apigenin 7-glucuronide), or the hydroquinone glycoside arbutin facilitated differentiation among species, sections, or groups. The noticeably large number of non-shared main constituents of *O. majorana*, including hydroquinone derivatives and a non-glycosylated flavonone, emphasises its distinctive status among the four species in section *Majorana* within this context. While the fundamental composition of the flavonoid spectrum in *Origanum* taxa seems largely influenced by evolutionary processes and genetic background, the significant variations in individual polyphenol content likely reflect the diverse site factors and sampling conditions previously noted (see also [59,63]). The production and accumulation of secondary metabolites are affected by several internal and external stimuli [64,65,66]. The pertinent literature regarding *Origanum* polyphenols has primarily concentrated on the influence of specific parameters, such as phenological stage and harvest time [30,31], on the content of a limited number of prominent compounds. The complex and dynamic interactions among secondary compound synthesis, environmental influences, developmental growth, and genetic determinants remain inadequately investigated. Integrative systems biology methodologies that amalgamate extensive biological data are essential for achieving a more profound and predictive comprehension of the intricate biological processes that govern the natural diversity of *Origanum* secondary compounds.

Various *Origanum* extracts or preparations, as well as the eight compounds quantified in this study, have been the subject of numerous investigations exploring their pharmacological activities and potential applications. Reported activities include strong antioxidative, antimicrobial, anti(retro)viral, anti-inflammatory, antidiabetic, antimutagenic, antigenotoxic, antitumor, antiarthritic, anti-Alzheimer and antistress effects; additionally neuroprotective, cardioprotective, hepatoprotective, osteoprotective, hypoglycaemic and wound healing properties have been described, as reviewed in [13,15,16,20,40]. Authors commonly conclude that the composition of secondary plant metabolites in raw materials is crucial for the therapeutic effect and efficacy of the resulting phytopharmaceuticals. The findings presented here can serve as a guide for the selection of appropriate raw materials for forthcoming pharmacological investigations and assist in the selection and cultivation of *Origanum* cultivars with distinct polyphenol profiles or elevated levels of a targeted compound. Focusing on the most utilised *Origanum* taxa, commercial *O. majorana*, *O. dubium*, *O. syriacum*, *O. onites*, and *O. vulgare*, their polyphenol chemotypes displayed distinct variations regarding the identified predominant phenolic acids (rosmarinic acid and salvianolic acid B) and main flavonoid glycosides (apigenin 6,8-di-glucopyranoside, luteolin 7-diglucuronide, luteolin 7-glucuronide, and apigenin 7-glucuronide). The chemotype of commercial *O. majorana* (cultivated in a temperate climate) demonstrated an average of 23 percent of the four main flavonoid glycosides (mean value 26 mg/g dry weight) and 40 percent of the two predominant phenolic acids (mean value 21 mg/g). The chemotype of wild *O. vulgare* subsp. *vulgare* exhibited 12 percent (13 mg/g) of the major flavonoid glycosides and 43 percent (23 mg/g) of the major phenolic acids. These two taxa can generally be regarded as the better sources of phenolic carbon acids. In the chemotype shared by *O. dubium*, *O. syriacum*, and *O. onites*, the ratio of the percentages of flavonoid glycosides to phenolic acids was inverted (*O. dubium* with 27 percent (69 mg/g) of the flavonoid glycosides and 17 percent (11 mg/g) of phenol carbon acids, *O. syriacum* with 32 percent (43 mg/g) of the flavonoid glycosides and 11 percent (4 mg/g) of phenol carbon acids) or more balanced (*O. onites* with 21 percent of the four flavonoid glycosides (13 mg/g) and 21 percent (9 mg/g) of phenol carbon acids). Specifically, *O. dubium* and *O. syriacum* are generally superior sources of apigenin and luteolin glycosides. Regarding specific compounds, there exists significant potential for the selection of genotypes particularly rich in arbutin (*O. majorana* wildtype, up to 99 mg/g dry weight in plants of this investigation), apigenin 6,8-di-glucopyranoside (*O. dubium*, up to 118 mg/g), rosmarinic acid (commercial *O. majorana*, up to 53 mg/g; *O. vulgare* subsp. *vulgare* up to 62 mg/g), and salvianolic acid B (wild *O. majorana*, up to 37 mg/g).

Twenty key components from a total of 122 were chosen to discuss the qualitative and quantitative variety of polyphenols in *Origanum* species. However, each of the over 100 minor components listed can contribute to synergistic effects that enhance the efficacy or spectrum of activity of *Origanum* plant extracts or augment the bioavailability of active extract components. Ref. [52] observed that minor variations in the composition of methanolic extracts of *O. vulgare* subsp. *hirtum* can result in distinct cytotoxic effects on model cancer cell lines. The intrinsic variability of *Origanum* plant material, coupled with the synergistic potential of complex plant extracts, underscores the pressing necessity for authentic and thoroughly standardised plant materials to ensure the reproducibility of pharmacological research results and to guarantee consistent biological activity for medicinal or prospective clinical applications.

## 4. Materials and Methods

### 4.1. Plant Material

A total of 657 plant samples of wild *O. majorana* L. (11 populations, 114 individual plants), commercial *O. majorana* varieties (13 seed origins, 186 individual plant samples), *O. dubium* Boiss. (7 wild populations, 73 individual plants), *O. syriacum* L. (syn. *Majorana syriaca* (L.) Kostel, 10 seed accessions and 1 commercial seed origin, 139 individual plant samples), *O. onites* L. (3 wild populations and 1 seed origin, 33 individual plants), *O. minutiflorum* O. Schwarz & P. H. Davis (4 wild populations, 48 individual plants), and *O. vulgare* subsp. *vulgare* (9 wild or cultivated populations, 64 individual plants) were analysed for this investigation. One part of the plant material was collected from native populations in Cyprus (late spring 2023), Austria, and Turkey (summer 2023). The geographical origin of the plant material from wild populations is shown in Figure S2, Supplementary Materials. With the exception of the Turkish populations of *O. onites* and *O. dubium*, the majority of the wild plants were harvested during full bloom. Wild-collected specimens were identified using the identification key from local floras [6,12,67]. The second part of the examined plant material originated from potted *O. majorana*, *O. syriacum* and *O. onites* specimens (greenhouse cultivation in winter, open land cultivation from early spring to late autumn). The seeds were acquired from various commercial suppliers (commercial *O. majorana*), sourced from the Botanical Garden Berlin Dahlem (*O. syriacum*) and the Israel Gene Bank (*O. syriacum*), or gathered during prior projects (*O. onites* and commercial *O. majorana*). The cultivated plants were mostly in full bloom at the time of harvest. The origin of all populations and seed accessions, along with the collection date and the number of analysed plant samples, are summarised in Table S1, Supplementary Materials. Voucher specimens of the wild Cypriot and cultivated populations are housed in the Herbarium of the Institute of Botany, University of Natural Resources and Life Sciences Vienna (WHB; voucher numbers 87868 to 87895). The voucher specimens of the Turkish populations are housed in the Herbarium of Div. of Industrial Crops, Department of Field Crops, Eskişehir Osmangazi University.

### 4.2. Sampling Procedure and Handling of Plant Material

Two to three representative shoots were selected from each sampled plant. As freeze-drying was not feasible due to the large number of wild-collected samples, the plant material was either air dried at ambient temperature (approx. 25–30 °C, wild populations) or dried in a drying cabinet (30 °C, greenhouse populations). The desiccated plant material was thereafter stored in cartons at ambient temperature in darkness. For analysis, all leaves of a branch and, if applicable, inflorescences were detached from the stems, while the stems and yellow or brown leaves were discarded. Leaves and inflorescences were stored individually in little paper bags. Prior to extraction, a representative sample of the coarsely crushed leaves or inflorescences was pulverised into a very fine, visually homogeneous powder (no coarse fragments observed) by utilising a ball mill (Pulverisette, Fritsch, Idar-Oberstein, Germany).

### 4.3. Extractions

Either 150 mg of finely ground leaf material or 100 mg of finely ground inflorescence material was extracted with 24 mL or 16 mL, respectively, of aqueous methanol (1:1) at room temperature, and placed for 30 min in an ultrasonic bath [24] (modified). The filtered extracts (syringe filters, 0.2 µm; Carl Roth, Karlsruhe, Germany) were aliquoted into HPLC vials and stored at −20 °C until analysis.

### 4.4. HPLC Analysis

High-Performance Liquid Chromatography (HPLC) analyses were conducted using a Shimadzu Nexera XR chromatograph (Shimadzu, Korneuburg, Austria), equipped with a controller (CBM-20A), a degasser (DGU-20A5R), a quaternary pump (LC-20ADXR), an autosampler (SIL-20AXR), a column oven (CTO-20AC) and a photodiode array detector (SPD-M20A). The software suite LabSolutions 5.97 (Shimadzu, Austria) was utilised for processing, data gathering, and data analysis. Separations were conducted using a Symmetry Shield RP18 column (5 µm, 4.6 × 250 mm; Waters, Vienna, Austria) coupled with a C18 guard column (ODS Octadecyl, 4 mm × 0.3 mm; Phenomenex, Aschaffenburg, Germany). A gradient elution was performed at a flow rate of 0.8 mL/min and an oven temperature of 25 °C using 0.25% (*v*/*v*) formic acid (Carl Roth, Germany) in acetonitrile (Carl Roth, Germany) and methanol (Carl Roth, Germany) (60:40) [solvent A] and 0.25% (*v*/*v*) formic acid in Milli-Q-water [solvent B]. The following gradient was used: min 0–7: 5–25% A in B (linear gradient), min 7–35: 25–35% A in B (linear gradient), min 35–40: 35–55% A in B (linear gradient), min 40–50: 70% A in B (isocratic), min 50–55: equilibrating to the initial 5% A in B (based on the protocol of [27], gradient slightly modified). Injection volume was 20 µL.

Following a comparative analysis of characteristic chromatograms of each *Origanum* species included, 122 significant and/or recurring peaks were defined (Table S2, Supplementary Materials) and further assessed. Peak detection was conducted at 280, 330 or 354 nm, depending on the specific UV/Vis absorption maximum of each listed peak. Identification and verification of trans-species occurrence of prominent compounds were conducted via evidence from the literature and by comparing retention times and UV spectra to those of available analytical standards or reference chromatography standards: apigenin (CAS registry number 303–98-0; Carl Roth, Germany), apigenin 6,8-di-glucopyranoside (CAS number 23666-13-9; Phytolab, Vestenbergsgreuth, Germany), apigenin 7-glucuronide (CAS number 29741-09-1; Phytolab, Germany), apigenin 7-glucoside (CAS number 578-74-5; Merck: Sigma-Aldrich, Vienna, Austria), apigenin 8-glucoside (CAS number 3681-93-4, Merck: Sigma-Aldrich, Austria), arbutin (CAS number 497-76-7; Carl Roth, Germany), blumeatin (CAS number 118024-26-3; Merck: Sigma-Aldrich, Austria), caffeic acid (CAS number 331-39-5; Merck: Sigma-Aldrich, Austria), carvacrol (CAS number 499-75-2; Carl Roth, Germany), catechin (CAS number 154-23-4; Merck: Sigma-Aldrich, Austria), chlorogenic acid (CAS number 327-97-9; Merck: Sigma-Aldrich, Austria), epicatechin (CAS number 490-46-0; Merck: Sigma-Aldrich, Austria), eriodictyol (CAS number 4049-38-1; Merck: Sigma-Aldrich, Austria), ferulic acid (CAS number 537-98-4; Merck: Sigma-Aldrich, Austria), gallic acid (CAS number 149-91-7; Merck: Sigma-Aldrich, Austria), hesperetin 7-rutinoside (CAS number 520-26-3; Merck: Sigma-Aldrich, Austria), hydroquinone (CAS number 123-31-9; Merck: Sigma-Aldrich, Austria), lithospermic acid A (CAS number 28831-65-4; Phytolab, Germany), luteolin (CAS number 491-70-3; Merck: Sigma-Aldrich, Austria), luteolin 7-diglucuronide (CAS number 96400-45-2; Phytolab, Germany), luteolin-7-O-glucuronide (CAS number 29741-10-4; Phytolab, Germany), naringenin (CAS number 67604-48-2; Merck: Sigma-Aldrich, Austria), naringenin 7-neohesperidoside (CAS number 10236-47-2; Merck: Sigma-Aldrich, Austria), protocatechuic acid (CAS number 99-50-3; Phytolab, Germany), quercetin (CAS number 849061-97-8; Merck: Sigma-Aldrich, Austria), quercetin 3-rhamnoside (CAS number 522-12-3; Merck: Sigma-Aldrich, Austria), rosmarinic acid (CAS number 20283-92-5; Merck: Sigma-Aldrich, Austria), salvianolic acid A (CAS number 96574-01-5; Phytolab, Germany), salvianolic acid B (CAS number 121521-90-2; Merck: Sigma-Aldrich, Austria), salvianolic acid C (CAS number 115841-09-3; Phytolab, Germany), and thymol (CAS number: 89-83-8; Carl Roth, Germany). The clear identification of minor peaks was sometimes obstructed by slight retention time variations during the analytical series, overlapping peak areas and unrepresentative UV spectra of smaller peaks.

The quantification of arbutin, apigenin 6,8-di-glucopyranoside, luteolin 7-O-diglucuronide, luteolin 7-O-glucuronide, rosmarinic acid, apigenin 7-O-glucuronide, salvianolic acid B, and blumeatin was conducted by comparison with the respective external standards. Calibration curves were constructed from six concentration levels. For each concentration, three independent dilutions were prepared, and each dilution was injected twice, yielding six measured injections per calibration point. Calibration parameters (equation, *r*^2^, LOD, and LOQ) are provided in Table S3, Supplementary Materials. Quantification was expressed as milligram per gram dry weight (mg/g dry wt).

Complete raw data (peak area table including 1185 samples and 122 peaks, table with quantified values of eight main components, calibration curves, UV spectra of main components and reference standards used for quantification) for the experiments have been deposited in Zenodo [68].

### 4.5. Statistics

Statistical analyses (including basic statistical parameters, analysis of variance (ANOVA), Tukey’s honestly significant difference (HSD) test, heatmap, and principal component analysis (PCA)) were conducted and visualised by RStudio 2024.09.01 [69] along with the following packages: agricolae, circlize, corrplot, complexHeatmap, dendextend, factoextra, FactoMiner, Hmisc, multcomp View, PerformanceAnalytics.

## Figures and Tables

**Table 1:** HPLC retention times (RT), peak ID, ʎ_max_ (nm), identification (n.i. = not identified) and relevance of the 20 major components (mean relative area % > 3 in ≥ 1 taxon). M = mean relative area %, SD = standard deviation. Taxa: maj = *O. majorana*, cmaj = commercial *O. majorana*, dub = *O. dubium*, syr = *O. syriacum*, oni = *O. onites*, min = *O. minutiflorum*, vul = *O. vulgare* subsp. *vulgare*. Species order reflects relatedness to *O. majorana*. Mean values > 5% are in bold. ^(1)^ confirmed with reference standard; ^(2)^ tentative ID based on literature. Compounds used for PCA are marked \*.

				maj		cmaj		dub		syr		oni		min		vul	
RT	Peak	ʎ_max_	Proposed Compound	M	SD	M	SD	M	SD	M	SD	M	SD	M	SD	M	SD
6.6	007	229/282	Arbutin *^(1)^	**9.0**	5.2	**7.2**	4.1	1.7	2.3	0.0	0.2	0.0	0.0	0.0	0.0	0.0	0.0
13.5	024	271/336	Apigenin 6,8-di-glucopyranoside *^(1)^	4.1	1.6	2.7	1.6	**9.5**	4.1	**7.4**	3.1	0.6	0.5	**7.9**	3.1	0.1	0.6
20.3	038	253/348	Luteolin 7-diglucuronide *^(1)^	0.2	0.1	0.1	0.1	0.0	0.1	0.2	2.2	0.8	0.6	**6.6**	3.5	**9.3**	6.1
21.3	040	222/263	n.i. *	0.0	0.0	0.0	0.0	0.0	0.1	0.0	0.4	0.0	0.0	0.0	0.0	**15.5**	7.3
23.4	044a	280/343	Flavonoid *^(2)^	0.0	0.0	0.0	0.0	0.2	0.3	0.0	0.1	**3.6**	2.1	0.7	0.8	0.0	0.0
24.0	045a	285/345	Flavonoid/Flavone *^(2)^	0.0	0.0	0.0	0.1	0.2	0.3	0.7	0.7	0.0	0.0	**6.4**	4.4	0.3	0.9
28.6	053	288	n.i. *	3.1	1.5	0.5	0.7	1.4	0.9	1.4	1.1	1.8	1.6	0.0	0.0	0.0	0.0
30.2	056	266/345	Luteolin 7-glucuronide ^(1)^	**9.2**	4.5	**12.7**	6.8	**7.2**	5.0	**9.0**	6.3	**11.2**	4.2	2.8	1.9	1.8	2.5
32.2	058	338	n.i. *	0.0	0.0	0.0	0.0	0.0	0.0	0.0	0.0	0.0	0.0	**6.1**	3.6	2.9	3.5
35.9	062	330	Rosmarinic acid *^(1)^	**18.9**	8.5	**37.2**	12.1	**15.1**	7.6	**11.2**	5.9	**21.0**	6.6	**12.0**	6.4	**42.9**	12.6
38.7	065	267/337	Apigenin 7-glucuronide ^(1)^	**6.9**	2.4	**7.0**	2.8	**9.9**	7.2	**14.9**	8.1	**8.3**	6.1	4.7	3.2	0.8	1.0
42.4	077	325	n.i.	0.4	0.5	3.3	3.3	2.6	3.2	4.3	2.6	0.2	0.4	0.9	1.0	0.3	0.6
42.5	077a	325	n.i.	0.3	0.5	**5.0**	5.0	4.3	5.3	**6.6**	4.0	0.3	0.4	1.8	2.7	0.6	0.9
43.1	080	287/309	Salvianolic acid B *^(1)^	**7.6**	3.5	2.7	3.7	1.6	1.3	0.1	0.3	0.3	0.7	0.2	0.3	0.4	1.1
43.5	083a	262/319	Globoidnan A *^(2)^	0.0	0.0	0.0	0.2	0.0	0.0	1.8	1.4	0.0	0.0	4.6	3.7	3.1	3.1
44.6	088	283/343	Flavonoid/Flavone *^(2)^	4.0	1.7	0.9	0.7	0.3	0.5	0.6	0.4	1.8	1.5	1.0	2.1	0.1	0.2
45.4	095	289/344	Flavonoid/Flavanone *^(2)^	**7.1**	2.3	1.5	1.2	1.3	1.2	1.0	0.7	3.1	2.1	0.7	0.8	0.1	0.1
45.8	097	287	Blumeatin *^(1)^	**5.7**	2.9	1.6	1.1	1.1	1.1	0.3	0.3	1.2	0.7	0.0	0.1	0.0	0.0
49.0	109	275	Carvacrol *^(1)^	0.0	0.0	0.0	0.0	**21.1**	11.7	**10.8**	13.2	**18.7**	4.4	**29.4**	9.5	0.2	1.9
49.6	110a	276	Thymol ^(1)^	0.0	0.0	0.0	0.4	1.6	6.2	**15.2**	14.5	0.0	0.1	0.0	0.0	0.0	0.0

## Data Availability

All data supporting the findings of this study have been deposited in Zenodo (https://zenodo.org/ (accessed on 15 September 2025)) and are available under the DOI 10.5281/zenodo.19002002 [68].

## Supplementary Materials

The following supporting information can be downloaded at: https://www.mdpi.com/article/10.3390/molecules31091531/s1, Table S1: Origin and collection details of analysed populations and seed accessions. Columns: population ID, number of plants analysed (n), collection date, species, latitude, longitude, geographical or material origin; Table S2: Complete peak table. Columns: peak number (Peak), RT (min), detection ʎ (nm), peak ID, proposed compound (n.i. = not identified). Mean relative area % (M) and standard deviation (SD). Taxa: maj = *O. majorana*, cmaj = commercial *O. majorana*, dub = *O. dubium*, syr = *O. syriacum*, oni = *O. onites*, min = *O. minutiflorum*, vul = *O. vulgare* subsp. *vulgare*. Major components (mean > 3%) are in bold and shaded. Variables with highest discriminatory power (top 30 from first four PCA dimensions) are marked \*; Table S3: Calibration parameters for quantified standards. Columns: compound, calibration equation, *r*^2^, concentration range (µg/µL), number of calibration points, number of independent preparations per calibration level, number of injections per preparation, number of injections per calibration level, LOD (µg/µL) and LOQ (µg/µL); Figure S1: Comparison of compound contents (mg/g dry wt) in leaves vs. inflorescences. Panels: (**A**) arbutin, (**B**) apigenin 6,8-di-glucopyranoside (syn. vicenin II), (**C**) luteolin 7-diglucuronide, (**D**) luteolin 7-glucuronide, (**E**) rosmarinic acid, (**F**) apigenin 7-glucuronide, (**G**) salvianolic acid B, and (**H**) blumeatin. Taxa: maj = *O. majorana*, cmaj = commercial *O. majorana*, dub = *O. dubium*, syr = *O. syriacum*, oni = *O. onites*, min = *O. minutiflorum*, vul = *O. vulgare* subsp. *vulgare*; Figure S2: Geographical origin of wild collections and seed accessions. Colour coding: Austrian *O. vulgare* subsp. *vulgare* (white), Turkish *O. onites* (turquoise), Turkish *O. minutiflorum* (dark-blue), Turkish and Cypriot *O. dubium* (green), Cypriot *O. majorana* (red), Israeli *O. syriacum* seed accessions (yellow). See Table S1 for coordinates and collection details. Map compiled with Google Earth (https://earth.google.com/web/ (accessed on 15 September 2025)).
