# Supplementary figures and images for: Polyphenol Diversity and Chemotype Variation in Origanum majorana and Related Species: Implications for Chemotaxonomic Differentiation, Standardisation and Genotype Selection
Source: Molecules. 2026 May 5;31(9):1531. doi: 10.3390/molecules31091531 (PMC13164803; doi:10.3390/molecules31091531)

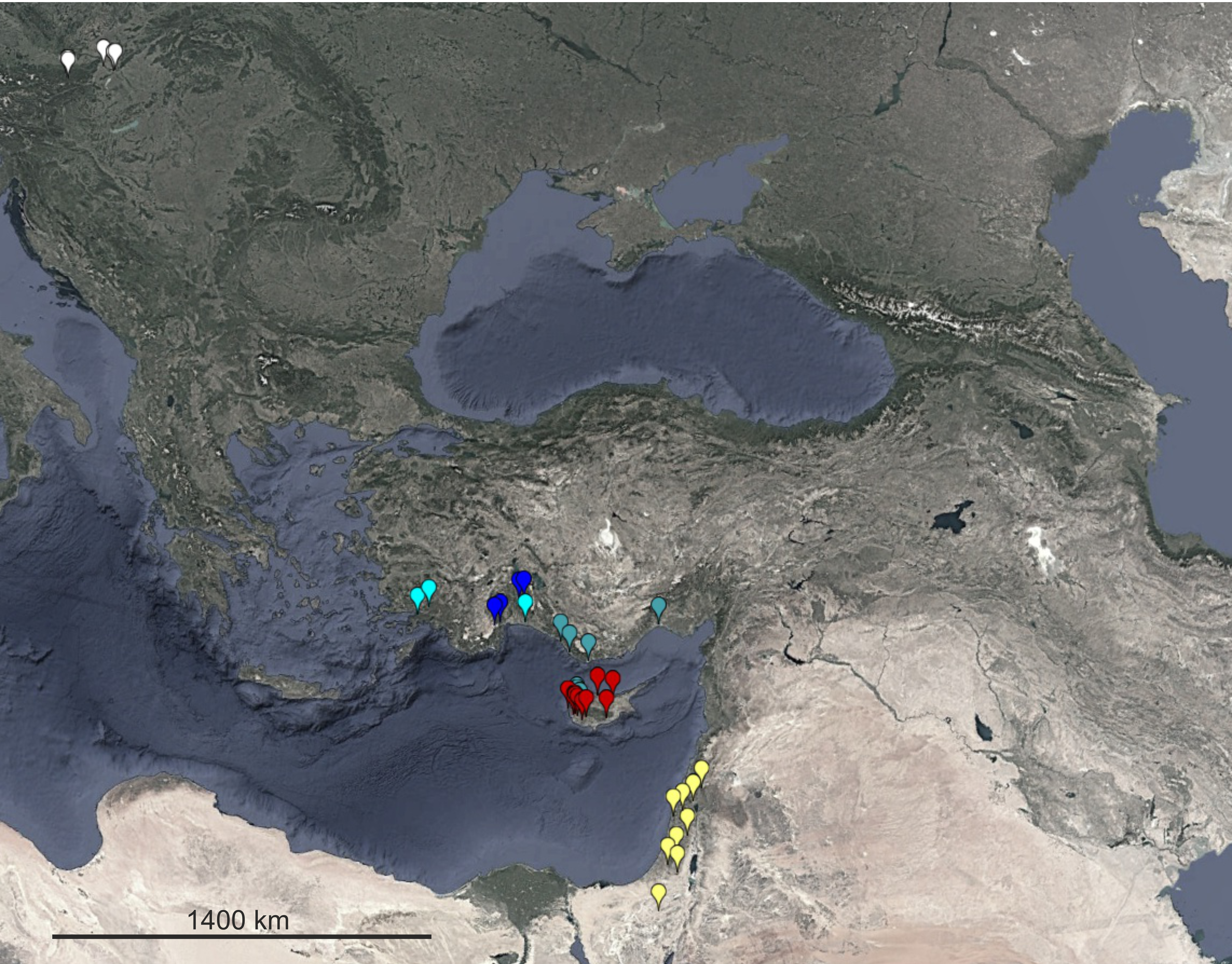

Supplement: Supplementary file 1 [file molecules-31-01531-s001.zip › SupplMaterials_FigureS2_Accessions.png]

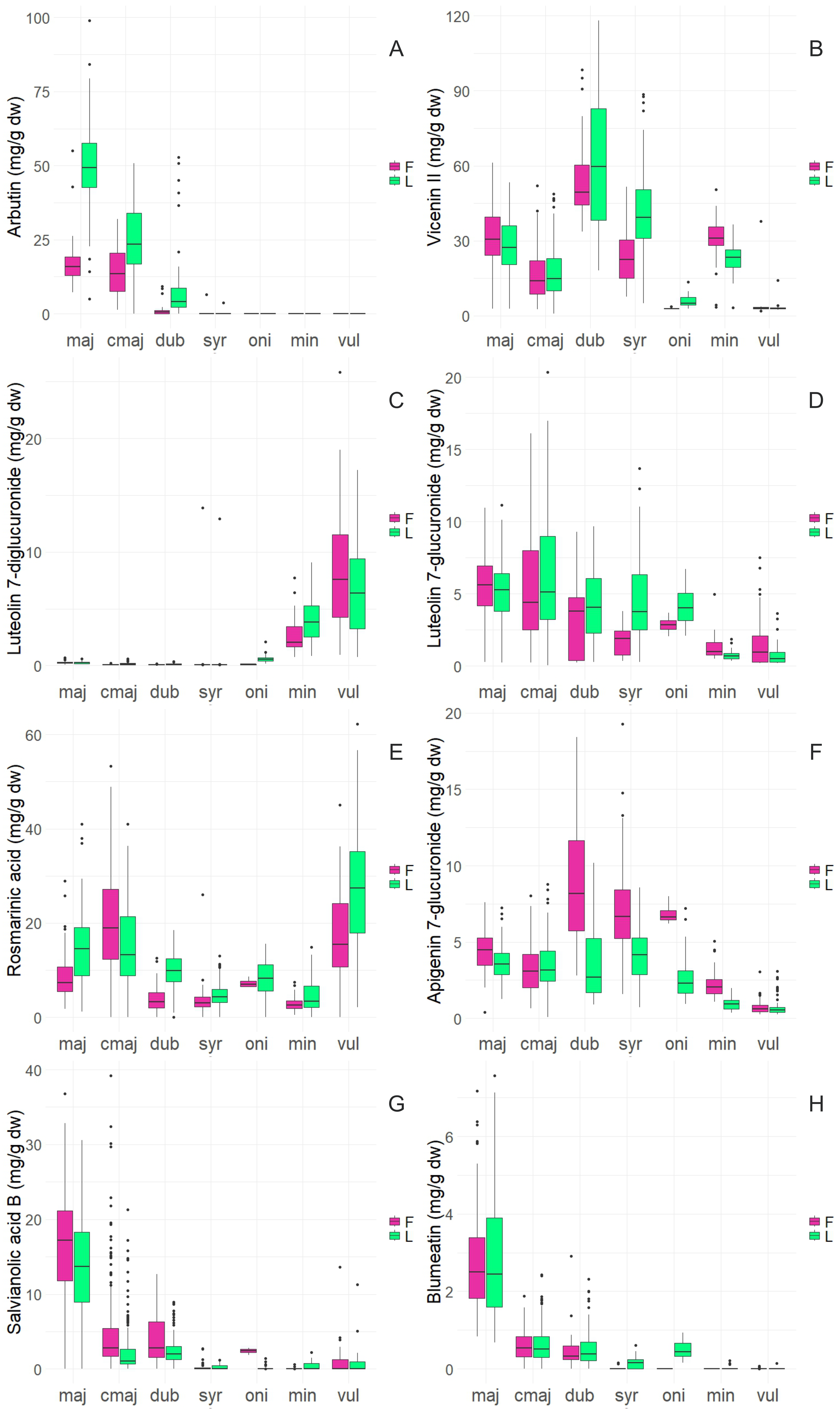

Supplement: Supplementary file 1 [file molecules-31-01531-s001.zip › SupplMaterials_FigureS1_QuantL-F.png]
